# Hydrodynamic Radii of Ranibizumab, Aflibercept and Bevacizumab Measured by Time-Resolved Phosphorescence Anisotropy

**DOI:** 10.1007/s11095-016-1940-2

**Published:** 2016-05-25

**Authors:** Liisa M. Hirvonen, Gilbert O. Fruhwirth, Nishanthan Srikantha, Matthew J. Barber, James E. Neffendorf, Klaus Suhling, Timothy L. Jackson

**Affiliations:** Department of Physics, King’s College London, Strand, London, WC2R 2LS UK; Department of Imaging Chemistry and Biology, Division of Imaging Sciences and Biomedical Engineering, King’s College London, St. Thomas’ Hospital, Lambeth Wing, London, SE1 7EH UK; School of Medicine, King’s College London, London, SE5 9RS UK; Department of Ophthalmology, King’s College Hospital, London, SE5 9RS UK

**Keywords:** fluorescence, hydrodynamic radius, phosphorescence, rotational diffusion, time-resolved anisotropy

## Abstract

**Purpose:**

To measure the hydrodynamic radii of intravitreal anti-VEGF drugs ranibizumab, aflibercept and bevacizumab with *μ*s time-resolved phosphorescence anisotropy.

**Methods:**

Ruthenium-based dye Ru(bpy)_2_(mcbpy − O − Su − ester)(PF_6_)_2_, whose lifetime of several hundred nanoseconds is comparable to the rotational correlation time of these drugs in buffer, was used as a label. The hydrodynamic radii were calculated from the rotational correlation times of the Ru(bpy)_2_(mcbpy − O − Su − ester)(PF_6_)_2_-labelled drugs obtained with time-resolved phosphorescence anisotropy measurements in buffer/glycerol solutions of varying viscosity.

**Results:**

The measured radii of 2.76±0.04 nm for ranibizumab, 3.70±0.03 nm for aflibercept and 4.58±0.01 nm for bevacizumab agree with calculations based on molecular weight and other experimental measurements.

**Conclusions:**

Time-resolved phosphorescence anisotropy is a relatively simple and straightforward method that allows experimental measurement of the hydrodynamic radius of individual proteins, and is superior to theoretical calculations which cannot give the required accuracy for a particular protein.

**Electronic supplementary material:**

The online version of this article (doi:10.1007/s11095-016-1940-2) contains supplementary material, which is available to authorized users.

## Introduction

There are many diseases that manifest in the posterior segment of the eye. These include age-related macular degeneration (AMD), retinal vein occlusion, and diabetic retinopathy and maculopathy. Together they account for the majority of blind registrations in the developed world (1). Many of these diseases are treated with regular injections of drugs into the vitreous cavity, with the inconvenience of regular clinic review, cost of repeated injection, discomfort, and small but repeated risks of complications (2). Given the many downsides of regular intravitreal injections, the drug industry is actively investigating novel methods of delivering drugs to the posterior segment, including sustained release intravitreal devices (3), transscleral drug delivery (4–6), topical drug delivery (eye drops) (7), oral (8) and others such as iontophoresis (9).

Topical drug delivery has many potential advantages, including self-administration, reduced cost, sustained drug levels, potentially fewer clinic visits, and the elimination of the risks associated with eye injections. Whilst desirable, topical drug delivery to the posterior segment is greatly impeded by the external ocular barriers to diffusion. This is compounded by the fact that many of the drugs used to treat posterior segment disease have a high molecular weight (MW), including ranibizumab (Lucentis®, 48 kDa), aflibercept (Eylea®, 97 kDa), and bevacizumab (Avastin®, 150 kDa).

Many factors, including the molecular size, charge, and three-dimensional structure in the given environment, will influence how intravitreal drugs cross the vitreous, and retina, to reach diseased macular and choroidal tissue (10–12). It is well known that increasing MW reduces diffusion across biological tissue (13–15), and other studies have shown that the molecular radius is a better predictor of tissue penetration than MW (4,5,15–18).

It is possible to estimate the radius of a protein from the MW. Erickson uses the fact that all proteins have approximately the same density, 1.37 g/cm^3^, to calculate the protein volume from the MW (19). Assuming a smooth spherical shape, this yields a minimum possible radius

1 $$ {R}_{min}\left(\mathrm{nm}\right)=0.066\cdot {\mathrm{MW}}^{1/3} $$

However, proteins have a rough surface, are often not perfectly spherical, and their charge can affect their diffusion in solution. The hydrodynamic radius *R*_*h*_, defined as the radius of a hard sphere that diffuses at the same rate as the solute, takes these effects into account. The hydrodynamic radius is important in predicting transretinal penetration (5,20).

Small-angle scattering studies using X-rays (SAXS) or neutrons (SANS) (21) as well as dynamic light scattering (DLS) (22,23) and nuclear magnetic resonance (NMR) techniques (24) have been used for measuring *R*_*h*_. Empirical relationships have been defined between *R*_*h*_ and the number of amino acids *N* (related to the MW by $N=\frac{MW}{110\kern0.5em  Da}$), for example, by Wilkins *et al.* (24)

2 $$ {R}_h^W\left(\mathring{\mathrm{A}} \right)=4.75\cdot {N}^{0.29} $$

and Dill *et al.* (25)

3 $$ {R}_h^D\left(\mathring{\mathrm{A}} \right)=1.45\cdot \left(2.24\cdot {N}^{0.392}\right)=3.248\cdot {N}^{0.392} $$

These formulas were obtained by global analysis of hundreds of proteins, and fitting to a scatter plot of *R*_*h*_ against MW. While they give a good indication of the expected size, there is a big variance in the measured *R*_*h*_ as a function of MW. This can be explained by the deviation of these models from the protein’s actual properties, which are due to molecular shape, charge and surface roughness.

Time-resolved fluorescence or phosphorescence anisotropy measurements can determine the molecule’s Brownian rotational mobility which depends on the molecular volume and the viscosity of the environment surrounding the molecule (26). The sample is labelled with a fluorescent or phosphorescent dye, and excited with a pulse of polarised light. The fluorescence or phosphorescence is collected in parallel and perpendicular polarisation directions as a function of time. The anisotropy *r(t)* of a molecule undergoing Brownian rotational diffusion in solution can be obtained from the measured intensities *I*_∥_ and *I*_⊥_ by

4 $$ r(t)=\frac{I{(t)}_{\parallel }-GI{(t)}_{\perp }}{I{(t)}_{\parallel }+2GI{(t)}_{\perp }} $$

where *G* is a correction factor that compensates for different transmission and detection efficiencies in the parallel and the perpendicular directions (27). If the sample solution contains spherical molecules of homogeneous size, the anisotropy decay follows a single-exponential function

5 $$ r(t)={r}_0\cdot {e}^{-\frac{t}{\phi }} $$

where *r*_0_ is the initial anisotropy at *t* = 0 and *ϕ* is the rotational correlation time. If the rotating unit is not spherical, a more complex multi-exponential model is required (28). *ϕ* can thus be obtained by fitting Eq. 5 (or the more complex model) to the experimental anisotropy decay. *ϕ* is related to the volume *V*, and thus the effective radius, of the rotating molecule by the Stokes-Einstein-Debye equation (29)

6 $$ \phi =\frac{\eta V}{kT} $$

where *η* is the solvent viscosity, *k* is the Boltzmann constant and *T* is the absolute temperature. Thus a plot of the rotational correlation time *ϕ versus* the viscosity *η* should be a straight line through the origin. The gradient should be *V*/*kT*, and thus yield the volume of the rotating unit. Any possible aggregation or preferential hydration would cause a deviation of the graph from the linear model.

Although time-resolved anisotropy measurements are a well established tool in molecular biology, only a few studies report applications in ophthalmology (8,30). In this study the effective hydrodynamic radii of three important posterior segment drugs—ranibizumab, aflibercept and bevacizumab—were measured by *μ*s time-resolved phosphorescence anisotropy and compared to radii calculated from the MW. Due to the large molecular weight of these drugs, short-lived nanosecond lifetime fluorescent dyes are not suitable for this study, as the fluorescence will have decayed by the time the drug has moved noticeably. Therefore, we used long-lifetime phosphorescence from a ruthenium-based dye (31) which allows us to measure the rotational diffusion of molecules with large molecular weights (32,33). A well-characterised protein, bovine serum albumin (BSA, MW = 66.5 kDa, *R*_*h*_ = 3.48 nm (34)), was also measured as a reference to validate our results.

## Method

### Reagents

Ranibizumab was purchased from Novartis (Frimley, UK) (Lucentis® 10 mg/ml) and bevacizumab from Roche (Welwyn Garden City, UK) (Avastin® 25 mg/ml). BSA was purchased from Sigma (Poole, UK) at the highest available purity. Aflibercept was purchased from Bayer plc (Newbury, UK) as a 40 mg/ml solution (Eylea®). All other reagents were of the highest available purity and were either from Sigma (Poole, UK), VWR (Lutterworth, UK), or Merck (Hoddesdon, UK) unless otherwise specified.

### Sample Preparation

All protein solutions were diluted with sterile phosphate buffered saline (PBS; Sigma; pH 7.4) to 5 mg/ml and then dialyzed twice against PBS pH 7.4 using D-Tube Midi Dialyzer units from Novagen (6–8 kD cut-off). Subsequently, the purity of the dialysed proteins was confirmed by standard SDS-PAGE. The proteins were then conjugated to the ruthenium dye. This was achieved by reacting the succinimidyl ester-modified dye, Bis(2,2′-bipyridine)-4′-methyl-4-carboxybipyridine-ruthenium N-succinimidyl ester-bis(hexafluorophosphate) (synonym, Ru(bpy)_2_(mcbpy − O − Su − ester)(PF_6_)_2_, Sigma-Aldrich, MW = 1 kDa) by using a succinimidyl ester-modified fluorophore with a short linker (Invitrogen, F6130). Conjugation reactions were performed in PBS adjusted with bicine buffer to pH 8.6 at 2 mg/ml protein concentration with the activated fluorophore ester being used in excess (2-fold and 3.5-fold for proteins >60 kD and ranibizumab, respectively). The reaction was stopped after 2 h and conjugated proteins were separated from remaining free dye by size exclusion chromatography (two times via 7 kD cut-off Zeba® spin columns (Thermo Fisher, UK)). Using this method we generated the various proteins conjugated to the fluorophore in the following dye:protein ratios: ranibizumab 1.1:1; BSA 1.2:1; aflibercept 1.8:1; bevacizumab 1.3:1, with concentrations of 47 *μ*M, 75 *μ*M, 56 *μ*M and 30 *μ*M, respectively. Azide (2.0 mM) was added to all dye-conjugated drugs to protect them from microbial deterioration.

The isoelectric point (pI) is the pH at which a molecule carries no net electrical charge, and is a major factor affecting protein stability in solution. It is an intrinsic property of any individual protein under the given conditions, and is the point of least stability in solution. The isoelectric point of BSA at 25^∘^C is pH = 4.7 (35). The theoretical isoelectric point of aflibercept (Eylea) as calculated using ExPASy is 8.2. (web.expasy.org) The isoelectric point of ranibizumab (Lucentis) and bevacizumab (Avastin) was previously determined to be 8.8 (36,37). Our experiments were performed at pH = 7.4, which is a good compromise and far away from the pI values of all compounds of interest and thus aggregation and precipitation are minimal under our conditions.

### Anisotropy Measurements

The Ru(bpy)_2_(mcbpy − O − Su − ester)(PF_6_)_2_-labelled drugs in buffer were mixed with glycerol in different proportions to produce solutions with different viscosities up to ∼70% volume fraction glycerol. 30 *μ*l of each mixture was placed in a multiwell plate with #1.5 coverslip glass bottom. The refractive index of each solution was measured with a refractometer (Bellingham+Stanley, UK) before and after the phosphorescence measurement and converted to viscosity using a function fitted to a conversion chart (38).

**Fig. 1:**
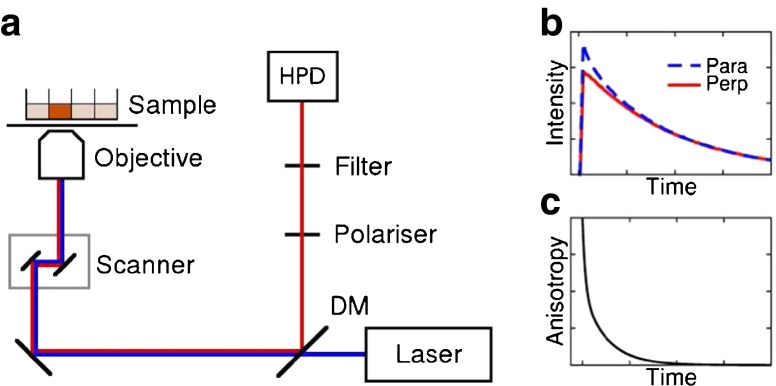
Illustration of the anisotropy measurements. (**a**) Schematic diagram of the experimental setup. The sample, i.e. the drug in solution, was placed on a confocal microscope stage and excited with a polarised pulsed laser. The phosphorescence from the sample was reflected from a dichroic mirror (DM) and recorded with a hybrid photodetector (HPD). (**b**) Schematic of the phosphorescence decays at parallel and perpendicular polarisation, from which the anisotropy (**c**) was calculated with Eq. **4**.

A schematic diagram of the experimental setup is shown in Fig. 1a. The anisotropy measurements were performed with a Leica TCS SP2, a standard confocal inverted microscope, equipped with a pulsed diode laser (PLP-10 470, Hamamatsu, Japan; optical pulse width 90 ps) that served as the excitation source (200 kHz repetition rate, 5 *μ*s time interval between pulses). The beam was focused in the middle of the well containing the sample solution with a 20× NA 0.5 air objective (Leica HC PL Fluotar). The emission was collected with the same objective through a 550 nm long-pass emission filter. A polariser was inserted in the emission path and parallel and perpendicular polarisation components of the fluorescence emission were recorded sequentially with a hybrid detector (HPM-100-40, Becker & Hickl GmbH, Berlin, Germany) connected to a time-correlated single photon counting (TCSPC) acquisition card (SPC 150, Becker & Hickl GmbH, Berlin, Germany). The measurement time window was 5 *μ*s, with 4096 time channels and a calibration of 1.22 ns/ch, and total data acquisition time of 30–60 min per data set. A test measurement with short data collection intervals confirms that bleaching does not cause problems with the measurements, see Section S1.

### Calculation of Hydrodynamic Radii

The anisotropies were calculated from the phosphorescence intensity decays measured in parallel and perpendicular polarisation directions with Eq. 4 (see Fig. 1b, c). The anisotropies contain a fast component in addition to the expected longer component and were fitted with gnuplot V4.6 (39) to a double-exponential function:

7 $$ y={A}_1{e}^{-\frac{t}{\phi_1}}+{A}_2{e}^{-\frac{t}{\phi_2}} $$

where *A*_1_ and *A*_2_ are the amplitudes and *ϕ*_1_ and *ϕ*_2_ the rotational correlation times of the two different components.

A plot of the longer rotational correlation time *ϕ*_2_ against viscosity yields a straight line, from which the hydrodynamic radius of the rotating unit can be calculated by combining the Stokes-Einstein-Debye equation (Eq. 6) with the equation for the volume of a sphere:

8 $$ {R}_h=\sqrt[3]{\frac{3kT}{4\pi}\frac{\phi_2}{\eta }} $$

where *k* is the Boltzmann constant, *T* is the absolute temperature and *ϕ*_2_/*η* is the gradient of the straight line.

## Results

**Fig. 2:**
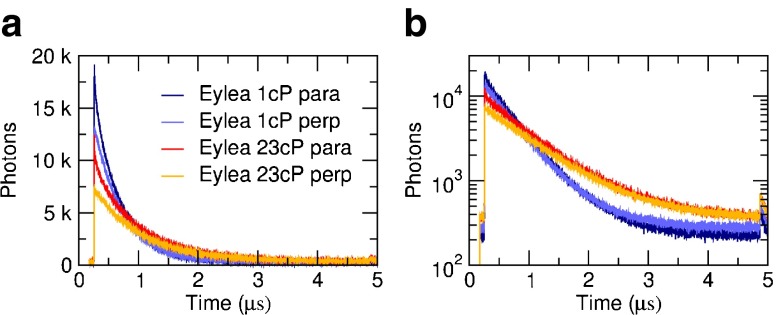
Measured raw phosphorescence decays for aflibercept at two different viscosities for parallel and perpendicular polarisation directions on a (**a**) linear and (**b**) semilogarithmic scale.

**Fig. 3:**
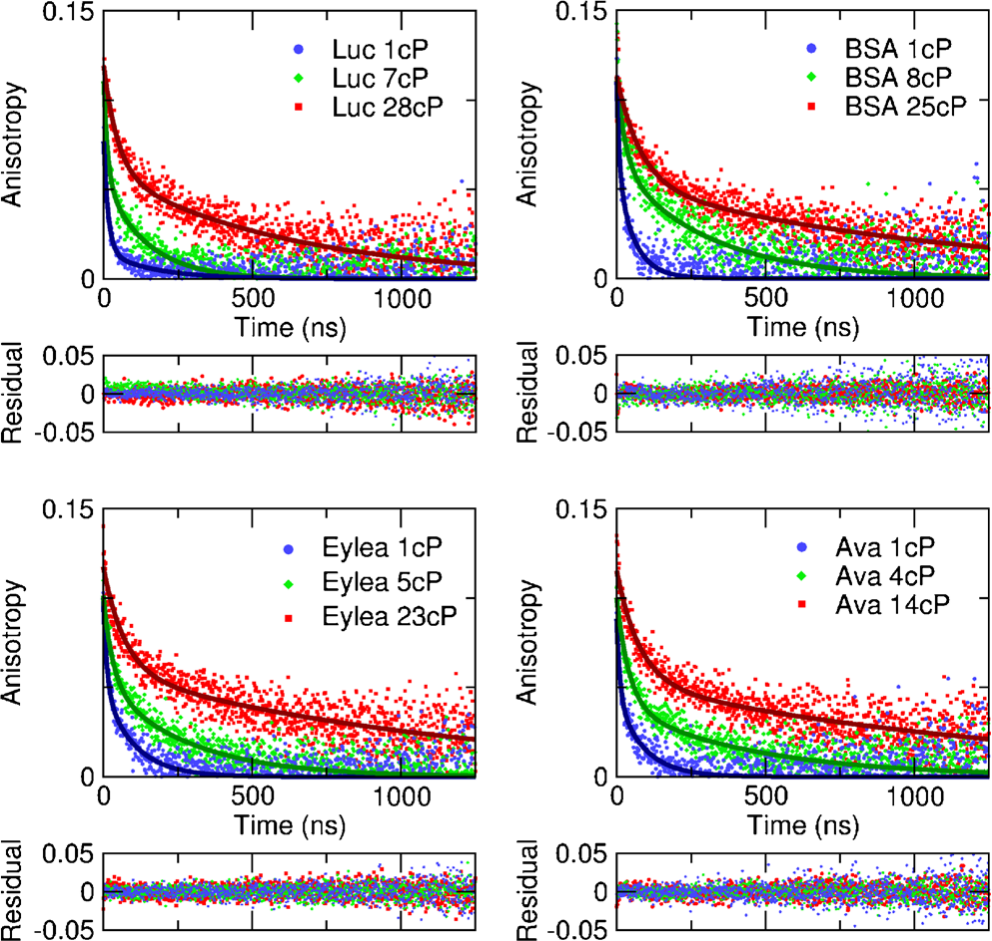
Example fits (*lines*) to measured anisotropy decays (*data points*) at different viscosities. The rotational correlation time increases with viscosity, and with the drug size. The decays were fitted with a double exponential function (Eq. **7**). The residuals are flat without systematic deviations, indicating a good fit.

The phosphorescence lifetime of the Ru(bpy)_2_(mcbpy − O − Su − ester)(PF_6_)_2_ compound increases with viscosity; examples of the measured raw decays are shown in Fig. 2. The time-resolved anisotropy decays were calculated according to Eq. 4, and representative examples are shown in Fig. 3. The anisotropy decay time (i.e. the rotational correlation time) increases with solvent viscosity for each drug, as expected (Fig. 3). A double-exponential fit to the anisotropy decay yields excellent fit results for all data sets; the fit results are consistent and largely independent of starting parameters and fitting range, and the residuals are flat without systematic deviations. See Fig S4 for goodness of fit parameters. Representative fits to three different viscosities for each drug are shown in Fig. 3.

**Fig. 4:**
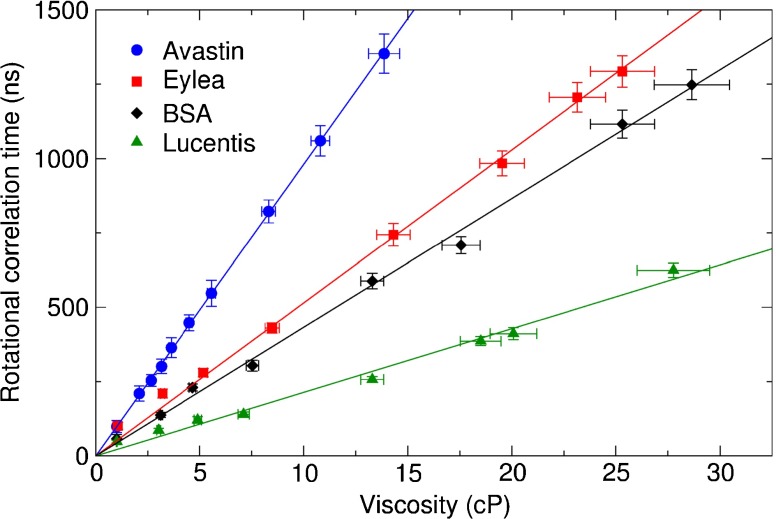
A plot of viscosity against rotational correlation time *ϕ*_2_ yields a straight line passing through the origin for all data sets, as expected from Eq. 6. The radius of the molecule can be calculated from the gradient with Eq. 8. The error in the x-axis was derived from the uncertainty in the measured refractive index, and the y-axis error from the uncertainty of the fit to the anisotropy using standard error propagation.

The shorter rotational correlation times, *ϕ*_1_, of the order of a few ns, were too fast to be measured accurately due to the long lifetime of the dye. The longer rotational correlation times, *ϕ*_2_, corresponding to the drug’s rotational diffusion were plotted against the viscosity, see Fig. 4. For each drug this yields a straight line passing through the origin, as expected from Eq. 6, whose gradient depends on the molecular volume. Gradients of 21.40±0.11 ns/cP for ranibizumab, 43.28±0.12 ns/cP for BSA, 51.47±0.12 ns/cP for aflibercept, and 98.09±0.04 ns/cP for bevacizumab were obtained by straight line fits according to Eq. 6 to the data sets using the least squares method. Using Eq. 8, this yields experimental hydrodynamic radii of 2.75±0.04 nm for ranibizumab, 3.49±0.03 nm for BSA, 3.70±0.03 nm for aflibercept, and 4.58±0.01 nm for bevacizumab. The theoretical radii of the drugs were also calculated according to Eqs. 1, 2 and 3. A summary of the calculated and measured hydrodynamic radii is shown in Table I.

**Table I:** Summary of Hydrodynamic Radii Measured Experimentally (R${}_{\mathbf{h}}^{\mathbf{meas}}$), Calculated with Empirical Formulas (R_**min**_, R${}_{\mathbf{h}}^{\mathbf{W}}$ and R${}_{\mathbf{h}}^{\mathbf{D}}$) and Reported DLS Results (R${}_{\mathbf{DLS}}^{\mathbf{Wen}}$ and R${}_{\mathbf{DLS}}^{\mathbf{Li}}$)

	Ranibizumab	BSA	Aflibercept	Bevacizumab	Equation	Reference
MW (kDa)	48	66.5	115	149		
R${}_{\mathrm{h}}^{\mathrm{meas}}$ (nm)	2.75±0.04	3.49±0.03	3.70±0.03	4.58±0.01	8	
R_min_ (nm)	2.40	2.67	3.21	3.50	1	(19)
R${}_{\mathrm{h}}^{\mathrm{W}}$ (nm)	2.77	3.04	3.52	3.85	2	(24)
R${}_{\mathrm{h}}^{\mathrm{D}}$ (nm)	3.52	4.00	4.87	5.49	3	(25)
R${}_{\mathrm{DLS}}^{\mathrm{Wen}}$ (nm)	4.2±0.3	5.4±0.1	–	6.3±0.1	–	(42)
R${}_{\mathrm{DLS}}^{\mathrm{Li}}$ (nm)	4.1	4.8	–	6.5	–	(36)

## Discussion

**Fig. 5:**
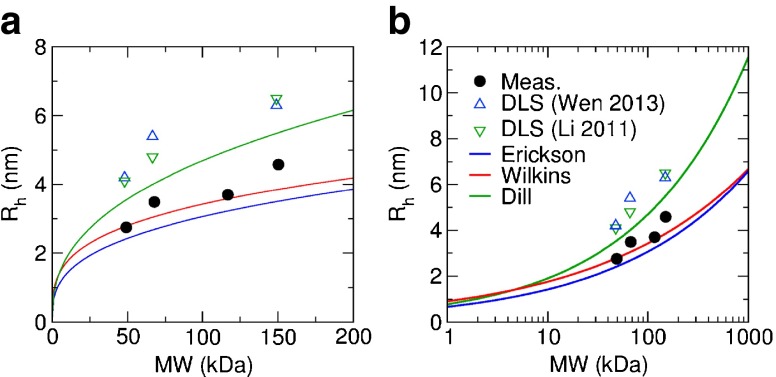
Comparison of hydrodynamic radii R_**h**_ measured experimentally (*black solid circles*) with radii calculated from empirical formulas (*lines*) and reported DLS results (hollow triangles) as a function of MW on a (**a**) linear and (**b**) semilogarithmic scale.

The radii obtained by anisotropy measurements are in the expected range and consistent with calculations based on MW, see Fig. 5, and our measured radius of 3.49±0.03 nm for the well-characterised protein BSA is in excellent agreement with the accepted value of 3.48 nm (34). While calculations based on the empirical formulas obtained from the analysis of thousands of proteins can give a good indication of the expected size, they cannot take into account the properties of specific molecules relating to surface roughness, shape, and the ionic charge which all affect the diffusion of the molecule.

Time-resolved anisotropy measurements depend on the volume of the compound undergoing Brownian rotational diffusion according to Eq. 6. The rotational correlation time depends on the cube of the radius, thus this technique is more sensitive to small changes in radius than methods where the radius depends on the measurement linearly, e.g. fluorescence correlation spectroscopy (FCS) and fluorescence recovery after photobleaching (FRAP) or DLS.

Time-resolved anisotropy measurements can also give information about the shape of the molecule via the model used to fit the decays, e.g. prolate and oblate ellipsoids of revolution can in principle be discerned with this approach (26,28). The anisotropies measured in this work are clearly double-exponential: besides the component corresponding to the rotation of the drug molecule, *ϕ*_2_, there is an additional fast component, *ϕ*_1_. However, this fast component cannot be attributed to non-spherical shape of the molecule; in case of an ellipsoid, the anisotropy decay is three-exponential, but the three exponentials are linked, and the fast component is too fast to fit this model (26,28). While it is too fast to be measured accurately with this method due to the long lifetime of the dye, the results indicate a size comparable to the dye molecule (MW = 1 kDa), and thus this fast component *ϕ*_1_ is most likely caused by the rotation of the dye molecule on its bond. Wilkins *et al.* report a similar fast component (24). Double-exponential fitting accounting for the fast component *ϕ*_1_ produces excellent fit results for our experimental data, where the Brownian rotational motion of the drugs is represented by a single slow component *ϕ*_2_, indicating that a spherical model for the molecules is appropriate for our data analysis.

DLS, based on the measurement of fluctuating scattered light intensity due to translational Brownian motion of the particles, is a well-established method for determining the size of small particles in solution, including macromolecular drugs (40). With the compounds much smaller than the wavelength of light, the light scattering is in the Rayleigh regime. To avoid multiple scattering, low solute concentrations (generally ≲1 mg/ml) should be used, and the solvent must have a different refractive index than the compound, otherwise they are invisible (41). DLS can be sensitive to dust particles, but does not require the compounds to be labelled with a dye, which simplifies the sample preparation. No considerations about the absorption spectrum of the dye, its emission spectrum and its lifetime and how to link it to the compound are needed. However, labelling the compound with a dye increases the specificity of the measurement, as only the labelled compound is measured, irrespective of its concentration, the refractive index or ionic strength of the solution, or other unlabelled solutes in the solution.

Wen *et al.* (42) report hydrodynamic radii of 4.2 nm for ranibizumab and 6.3 nm for bevacizumab using DLS, and 5.4 nm for BSA measured as a control, and Li *et al.* (36) 4.1, 6.5 and 4.8 nm for the same molecules, respectively. These results are slightly higher than estimates based on MW, and higher than our results obtained with time-resolved phosphorescence anisotropy. Possible reasons could include aggregation, multiple scattering or a larger solvent cage that is dragged around in translational diffusion.

SAXS and SANS are also popular methods for the size measurement of macromolecules. While they can be used with higher particle concentrations than DLS (∼1–100 mg/ml) and are applicable to a large MW range from a few kDa to hundreds of MDa, they have low resolution, and structural information can only be obtained through complex model building (22). Similar to DLS, SAXS and SANS measure scattering from unlabelled molecules which simplifies sample preparation but makes the results susceptible to artefacts arising from dust and other contamination in the sample solution, and makes these techniques impossible to be used with scattering media, such as tissue.

The measurement time interval of 5 *μ*s used in our experiments is ideal for measuring the rotational correlation times of these drugs with this ruthenium-based dye at low viscosities. The detection volume could go down to the focal spot of a scanning microscope operating at the optical diffraction limit, and femtoliter volumes could be probed (43,44).

Due to the strong dependence of the rotational correlation time on the molecular volume, see Eq. 6, anisotropy measurements can be used to study binding or cleavage. Using a genetically encoded probe with 13 ns fluorescence lifetime, LUMP, it was shown that steady-state anisotropy measurements could detect binding with CDC42 (32). DNA digestion has also been monitored using fluorescently labelled DNA, as indicated by a decrease in the anisotropy over time after addition of a DNA digesting enzyme (45). Moreover, when formulating drugs, by addition of other components, sometimes even other proteins, fluorescence or phosphorescence labelling before formulating would allow to specifically identify and measure the protein of interest within a mixture.

If the long lifetime probe labelling approach is combined with imaging, Phosphorescence Lifetime Imaging (PLIM) (31,46) can be performed, either with scanning confocal or wide-field microscopy. We have recently developed wide-field lifetime imaging approaches (47,48) that are ideally suited for measuring microsecond lifetimes, and could image several wells containing different drugs and/or different viscosity solutions simultaneously. If these microsecond-resolution wide-field time-correlated single photon counting approaches were to be combined with polarisation-resolved excitation and detection, one could perform time-resolved anisotropy imaging (49) on a microsecond time scale. This would benefit the measurement of similar or higher MW drugs in several wells of a multiwell plate simultaneously, and also enable imaging these drugs *in vitro*. Alternatively, this approach could be employed to map viscosity in cells (49,50).

## Conclusion

Anisotropy measurements are commonly used in biochemical applications to provide information on the volume of proteins, as well as the viscosity of their surrounding environment. We have shown that the hydrodynamic radii of anti-VEGF proteins ranibizumab, aflibercept and bevacizumab can be measured accurately using time-resolved anisotropy and a ruthenium-based dye with a phosphorescence decay time of hundreds of nanoseconds as a label. This relatively simple and straightforward approach allows the experimental measurement of the hydrodynamic radius of individual proteins. It is superior to theoretical estimations which cannot give the required accuracy for a particular protein. The measured phosphorescence anisotropy decays are double-exponentials, with a long component caused by the Brownian rotational diffusion of the drug molecule, and a short component due to the dye rotating on its bond. The measurement of well-characterised protein BSA as a reference confirms the validity of this approach. The experimental radii for BSA, ranibizumab, aflibercept and bevacizumab are in good agreement with the radii calculated from molecular weight and other experimental measurements.

## Electronic supplementary material

Below is the link to the electronic supplementary material.

ESM 1 (DOCX 18.8 kb)

ESM 2 (GIF 148 kb)

High resolution image (EPS 350 kb)

ESM 3 (GIF 12 kb)

High resolution image (EPS 233 kb)

ESM 4 (GIF 6 kb)

High resolution image (EPS 267 kb)

ESM 5 (GIF 31 kb)

High resolution image (EPS 107 kb)

## Abbreviations

AMD: Age-related macular degeneration
BSA: Bovine serum albumin
DLS: Dynamic light scattering
FCS: Fluorescence correlation spectroscopy
FRAP: Fluorescence recovery after photobleaching
MW: Molecular weight
NMR: Nuclear magnetic resonance
PBS: Phosphate buffered saline
PLIM: Phosphorescence lifetime imaging
SANS: Small-angle neutron scattering
SAXS: Small-angle x-ray scattering
VEGF: Vascular endothelial growth factor
