# Supplementary material for: Hydrodynamic Radii of Ranibizumab, Aflibercept and Bevacizumab Measured by Time-Resolved Phosphorescence Anisotropy
Source: Pharm Res. 2016 May 25;33:2025–32. doi: 10.1007/s11095-016-1940-2 (PMC4942501; doi:10.1007/s11095-016-1940-2)
Supplement: Supplementary file 1 — (DOCX 18.8 kb) [file 11095_2016_1940_MOESM1_ESM.docx]

**SUPPORTING INFORMATION**

**Hydrodynamic radii of ranibizumab, aflibercept and bevacizumab measured by time-resolved phosphorescence anisotropy**

# S1. Data collection – bleaching test

Although the illumination intensity used in these experiments is extremely low, an experiment was performed to confirm that the measurements are not affected by bleaching over the 30-60 min data collection period. Data was collected over an hour in 5-minute intervals, changing polarisation between 0 and 90 degrees every 5 minutes (i.e. measurement 1: 0 degrees 0-5 min, 90 degrees 5-10 min, measurement 2: 0 degrees 10-15 min, 90 degrees 15-20 min, etc.). The measured phosphorescence decays do not change systematically over time, see Fig S1.

The anisotropy was also calculated for the 6 measurement intervals (i.e. measurement 1: 0-10 min, measurement 2: 10-20 min, etc.), see Fig S2. The data is very noisy due to the low photon count at 5 min time intervals, and Fig S2 shows the moving average of 10 data points. The anisotropy does not show a systematic change over time.

Figure S1. BSA at 3.3 cP viscosity. The total data collection time was an hour, with the polarisation changed between 0 and 90 degrees every 5 minutes. (a) Data collected at 0 degrees, (b) data collected at 90 degrees, i.e. measurement 1: 0 degrees 0-5 min (graph (a)), 90 degrees 5-10 min (graph (b)), measurement 2: 0 degrees 10-15 min (graph (a)), 90 degrees 15-20 min (graph (b)), etc.)

Figure S2. Anisotropy (moving average of 10 data points) calculated for the different measurement periods in Fig S1 (i.e. measurement 1: 0-10 min, measurement 2: 10-20 min, etc.).

# S2. Phosphorescence lifetimes

The measured phosphorescence lifetimes are shown in Figure S3a as a function of viscosity. Figure S3b shows the lifetimes in a Stern-Volmer plot, with the inverse viscosity on the x-axis, which is qualitatively consistent with partial oxygen quenching of the phosphorescence described by a modified form of Stern-Volmer equation. [S1]

Figure S3. (a) Measured phosphorescence lifetimes $\boldsymbol{\tau}$ as a function of viscosity, and (b) a Stern-Volmer plot of $\boldsymbol{\tau}_{\boldsymbol{0}}\boldsymbol{/\tau}$ as a function of 1/viscosity.

# S3. Anisotropy – goodness of fit

Figure S4 shows the chi-square values (i.e. sum of squares of residuals) for the anisotropy decays.

Figure S4. Chi-square values (sum of squares of residuals) for the double-exponential fits to the measured anisotropies.

**References**

[S1] Lakowicz JR. Principles of fluorescence spectroscopy. 3rd ed. New York: Springer; 2006. p. 279–81
