# Supplementary figures and images for: Hydrodynamic Radii of Ranibizumab, Aflibercept and Bevacizumab Measured by Time-Resolved Phosphorescence Anisotropy
Source: Pharm Res. 2016 May 25;33:2025–32. doi: 10.1007/s11095-016-1940-2 (PMC4942501; doi:10.1007/s11095-016-1940-2)

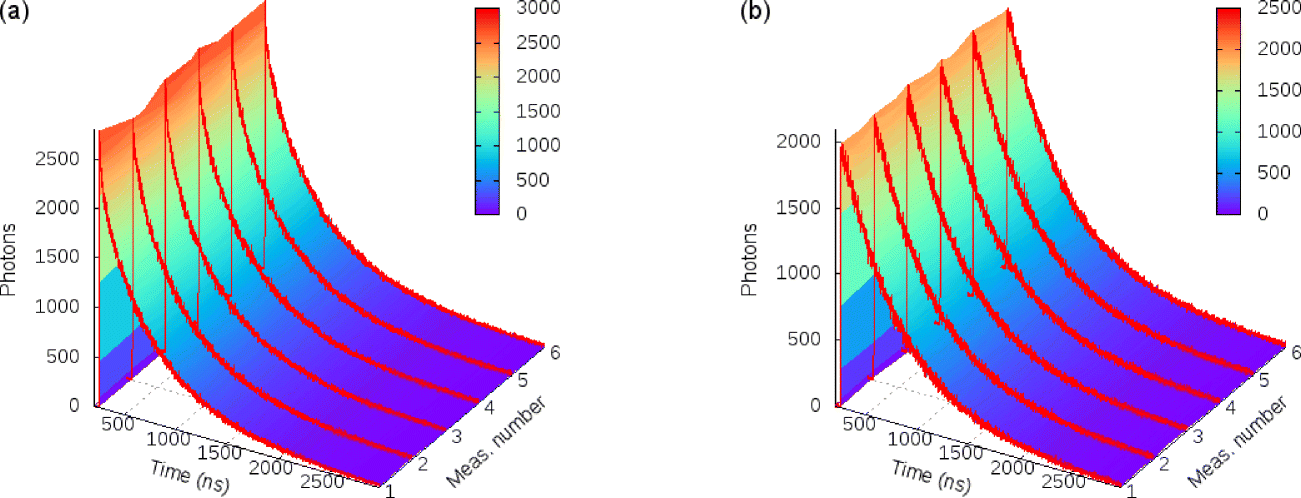

Supplement: Supplementary file 2 — (GIF 148 kb) [file 11095_2016_1940_Fig6_ESM.gif]

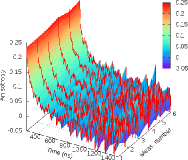

Supplement: Supplementary file 4 — (GIF 12 kb) [file 11095_2016_1940_Fig7_ESM.gif]

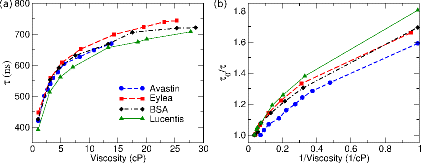

Supplement: Supplementary file 6 — (GIF 6 kb) [file 11095_2016_1940_Fig8_ESM.gif]

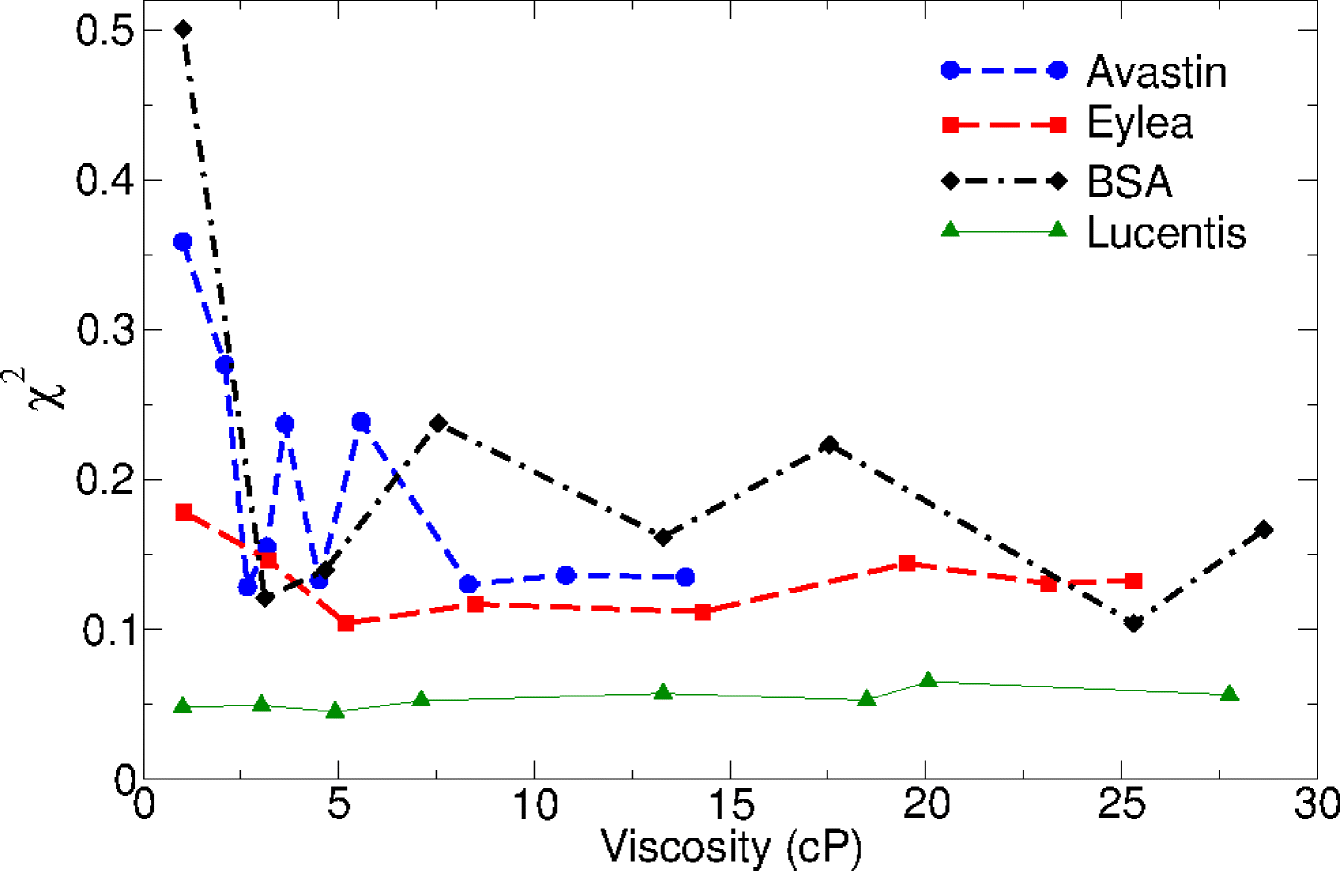

Supplement: Supplementary file 8 — (GIF 31 kb) [file 11095_2016_1940_Fig9_ESM.gif]
